# Delayed-onset flaccid paralysis related to west Nile virus reactivation following treatment with rituximab: a case report

**DOI:** 10.1186/1756-0500-7-852

**Published:** 2014-11-26

**Authors:** Asaf Honig, Dimitrios Karussis

**Affiliations:** Department of Neurology, Laboratory of Neuroimmunology and Agnes Ginges Center for Neurogenetics and Multiple Sclerosis, Hadassah-Hebrew University Hospital, Ein-Karem, Il-91120 Jerusalem, Israel

**Keywords:** West Nile virus, Acute flaccid paralysis, Rituximab, Delayed immune reaction, Poliomyelitis

## Abstract

**Background:**

Neurological manifestations of West Nile virus infection include meningitis, encephalitis and acute flaccid paralysis. Typically, West Nile virus-associated acute flaccid paralysis is characterized by acute and rapidly progressing limb weakness, occurring early in the course of the disease.

**Case presentation:**

We report a patient of Yemenite descent who developed West Nile virus-encephalitis and poliomyelitis two weeks following treatment with rituximab for B cell lymphoma, and delayed encephalitis with ascending demyelinating polyneuropathy 6 months later. Diagnosis of the first episode was based on a high West Nile virus copy number in the blood polymerase chain reaction. During the second episode the patient developed encephalitis and flaccid asymmetric quadriparesis, accompanied by high IgM anti-West Nile virus titers in the blood and cerebrospinal fluid.

**Conclusion:**

The delayed polyneuropathy post-West Nile virus infection and encephalitis/poliomyelitis may be related to reactivation of the virus or to a delayed autoimmune (post-infectious) process, possibly accelerated by the recovering B-cell humoral immunity, 6 months after treatment with rituximab. This case depicts the complexities of the immune responses and their reconstitution following monoclonal antibody treatment and the diversity of neurological syndromes associated with West Nile virus infection.

## Background

Only less than one percent of individuals infected by the West-Nile virus (WNV) develop neurological manifestations [1]. These include meningitis, encephalitis, and acute flaccid paralysis (AFP). Delayed flaccid paralysis/polyradiculitis has also been described several weeks following WNV-infection [2]. WNV anterior poliomyelitis usually occurs early in the course of the infection. Jackson *et al.*[2] recently described four atypical cases of WNV-poliomyelitis. In one of them the onset of poliomyelitis was delayed (several weeks following the initial infection) and three patients suffered from relapsing limb weakness following a period of clinical remission. The authors suggested that the delayed WNV-poliomyelitis could be explained by a chronic infection or delayed neuroinvasion. This is supported by the finding that WNV-ribonucleic acid may be detected in the urine of convalescent patients up to 7 years following infection [3]. However, attempts to grow the virus from the urine samples of these patients were not successful. Additional studies are necessary to determine the significance of this finding.

We describe here a patient who developed WNV-encephalitis and poliomyelitis two weeks following treatment with rituximab for B cell lymphoma, and delayed ascending demyelinating polyneuropathy 6 months later.

## Case representation

A 57 year- old- male, of Yemenite descent, was admitted to the Department of Neurology at Hadassah University Hospital due to encephalitis. B-cell lymphoma had been diagnosed one year earlier and after a year of management with cytotoxic medications, it was decided to start treatment with rituximab. Two weeks following initiation of rituximab, the patient suffered from high fever and confusion. During his hospitalization, he developed weakness in his right leg. The laboratory and microbiological evaluation was unremarkable, except for the blood-polymerase chain reaction (PCR), which was positive for WNV (examined 3 days after the initial presentation). The blood and cerebrospinal fluid (CSF) were negative for WNV antibodies, both IgG and IgM, possibly due to defective humoral responses caused by the treatment with rituximab. CSF analysis showed 9 white blood cells and an elevated protein level of 850 mg per liter. A diagnosis of WNV-encephalitis/poliomyelitis was made. The patient recovered from the encephalitis with a residual mild weakness in his right leg and a mild cognitive impairment.

Six months later, the patient was re-hospitalized due to vomiting, instability of gait and a fever of 38°C. An ascending paralysis developed gradually, starting symmetrically in the legs and involving (four days later) the hands.

Neurological examination revealed dysarthric speech, bilateral horizontal nystagmus, more evident to the right, weakness of the bulbar muscles, as well as the neck flexors, weak reflexes of the right hand but well-retained in the other limbs, and a right plantar extension reflex, accompanied by prominent frontal release signs. He had truncal ataxia and bilateral dysmetria. His mini-mental score was 27/30, with marked slowness of thinking.

The laboratory workup was unremarkable, excluding a sedimentation rate of 84.

Serological tests for mycoplasma, Rickettsia conorii, Salmonella typhi, HIV, HTLV and chlamydia were negative, as well as for anti-GM1 antibodies. A lumbar puncture revealed mild CSF pleocytosis (12 lymphocytes) and an elevated CSF protein level (625 mg/l). The serology for WNV showed consistently high titers of IgM anti-WNV antibodies, whereas the IgG anti-WNV antibodies remained negative. The PCR for WNV in the blood, CSF and urine was negative. A CSF-PCR for a panel of enteroviruses and microbiological tests for fungi, were all negative. Neuroimaging, (CT and MRI) of the brain and cervical spinal cord did not reveal any pathology. A bone marrow biopsy and PET-CT imaging did not show recurrence of the lymphoma.

The EMG tests showed prolonged motor latencies and slow conduction velocities, with dispersion in multiple nerves of the upper limbs and conduction block in the nerves of the legs, supporting the diagnosis of demyelinating polyneuropathy. Following treatment with 5 doses of intravenous immunoglobulins (IVIG) and a high dose of intravenous methylprednisolone (IVMP), there was remarkable improvement of the paralytic disease. The patient died two years following the reported events of unrelated causes.

## Conclusions

In our case, a delayed ascending demyelinating polyneuropathy with flaccid asymmetric quadriparesis developed 6 months following WNV-infection with encephalitis and, possibly, mild poliomyelitis (right leg paresis). Polyradiculitis has been reported up to few weeks following WNV-infection, but such a delayed onset is very unusual. Possible explanations for this recurrence (and expansion) of the neurological involvement caused by WNV may include persistent/chronic viral infection of the central and peripheral nervous systems or reactivation of the virus [3]) and secondary immune-mediated (post-viral) mechanisms. The latter may be related to the rebound of antibody reactivity during the recovery of the suppressed humoral immune responses, 6 months after treatment with rituximab. The high IgM anti-WNV antibody titers (which were negative initially) may support this possibility and indicate an acute, delayed immunological reaction.

Alternatively, the ascending polyneuropathy may represent Acute Inflammatory Demyelinating Polyneuropathy (AIDP) not necessarily directly caused by the WNV but possibly related to the treatment with rituximab. Development of AIDP and of autoimmune diseases following treatment with cytotoxic or immunosuppressive medications, including rituximab, has been reported in the literature [4, 5]. This paradoxical phenomenon depicts and underlines the intricate and delicate interactions inside the immune system network. Immunosuppression may occasionally affect predominantly the regulatory or suppressor lymphocytes and allow the expansion of otherwise suppressed auto-reactive lymphocytes, leading to the break of self tolerance and the development of autoimmune disease [6]. Specifically, in the case of monoclonal antibodies such as rituximab, the delayed development of autoimmune syndromes may be related to the reconstitution phase of the B-cells, which during their recovery may cause a strong rebound of antibody production, capable of breaking self tolerance. The negative CSF PCR for WNV is a puzzling finding that advocates against the possibility of a persistent infection. It thus seems more likely that the delayed neurological syndrome in our case was caused by an immune-mediated mechanism similar to that involved in the pathogenesis of other post-viral syndromes. Molecular mimicry plays the major role in the pathogenesis of such post-viral syndromes. A typical example of such an autoimmune disease is post-infectious AIDP, which is often caused by an immune response that initially targets Campylobacter jejuni [7].

The possibility that the polyneuropathy in our case was (directly or indirectly) related to the lymphoma is highly unlikely, due to the fact that there were no signs of reactivation of the disease in the PET-scan and no malignant lymphoma cells were found in the CSF (negative immunoglobulin gene rearrangement in the CSF). Also, anti- Hu antibodies, indicative of a paraneoplastic syndrome were not present.

The association of rituximab with WNV was reported by Levi *et al.*[8] in a patient who developed rapid, fulminant WNV meningoencephalitis 6 months following rituximab treatment and succumbed.

In summary, it seems likely that in the present case, the delayed flaccid paralysis and ascending polyneuritis, in the absence of evidence of chronic persistent neurological infection with WNV, was most probably caused by an immune reconstitution syndrome, similar to that described in HIV patients or following organ transplantation and possibly accelerated by the recovering B-cell humoral immunity, 6 months after treatment with rituximab.

## Consent

Written informed consent was obtained from the patient’s next of kin for publication of this Case Report and any accompanying images. A copy of the written consent is available for review by the Editor-in-Chief of this journal.

## Abbreviations

WNV: West Nile virus
AFP: Acute flaccid paralysis
CSF: Cerebrospinal fluid
PCR: Polymerase chain reaction.
IVIG: Intravenous immunoglobulins
IVMP: Intravenous methylprednisolone.
